# Lung ultrasound for etiological diagnosis of pneumonia in the emergency department: correlation with bronchoalveolar lavage results

**DOI:** 10.1186/s13089-025-00470-0

**Published:** 2025-11-27

**Authors:** Lorenzo Pelagatti, Iacopo Vannini, Francesca Mangani, Gian Maria Rossolini, Giovanni Volpicelli, Simone Vanni, Peiman Nazerian

**Affiliations:** 1 Emergency Department, Careggi University Hospital, Largo Brambilla 1, Florence, 50134 Italy; 2 Department of Experimental and Clinical Medicine, University of Florence, Florence, Italy; 3 Microbiology and Virology Unit, Careggi University Hospital, Florence, Italy; 4 Department of Medical and Surgery Science, Magna Graecia University, Catanzaro, Italy; 5 High Dependency Unit, Careggi University Hospital, Florence, Italy

**Keywords:** Lung ultrasound, Molecular syndromic panels, PCR, Bronchoalveolar lavage, Bronchoscopy

## Abstract

**Background:**

Pneumonia is the leading cause of death from infectious diseases worldwide. Lung ultrasound (LUS) is highly accurate for chest infections diagnosis, yet its correlation with causative pathogens remains unclear. Respiratory cultures, combined with molecular techniques represent the gold standard, achieving etiological diagnosis in 90–95% of cases. We compared LUS findings with bronchoalveolar lavage (BAL) sample analyses; to our knowledge, no prior studies have investigated this in the emergency department (ED).

**Materials and methods:**

Bronchoalveolar lavage (BAL)-LUS is a prospective observational non-profit study conducted in the ED, aiming to assess whether there is a correlation between the LUS sonographic appearance, assessed blindly across 12 lung fields, and the etiopathogenetic agent of pneumonia (bacterial and viral) detected with molecular syndromic panels (MSPs) and respiratory cultures obtained with BAL.

**Results:**

64 patients were enrolled (mean age 73.3 ± 14.6) with 11 diagnosed as viral pneumonia and 53 as bacterial pneumonia. Bacterial pneumonias were more commonly associated with consolidation (2.9 ± 2.2 vs. 1.5 ± 0.9, *p* < 0.01) and a higher incidence of pleural effusion (0.9 ± 1.3 vs. 0.3 ± 0.6, *p* < 0.01). Viral pneumonias were more often associated with interstitial syndrome (4.9 ± 3.3 vs. 0.5 ± 1.3, *p* < 0.01) and small subpleural consolidations (0.9 ± 1.8 vs. 0.2 ± 0.6, *p* = 0.01). The mean LUS score was significantly higher in bacterial than in viral pneumonia with a AUC of 0.81 (95% CI 0.68–0.93).

**Conclusions:**

Viral pneumonia is usually associated with interstitial syndrome and small subpleural consolidations; on the other hand, bacterial pneumonia is usually associated with consolidation, and pleural effusion.

**Supplementary Information:**

The online version contains supplementary material available at 10.1186/s13089-025-00470-0.

## Introduction

 Pneumonia remains the leading cause of death from infectious diseases worldwide, accounting for over 3.1 million deaths annually. It is the eighth leading cause of death overall and the most frequent trigger of sepsis, with mortality being highest among hospitalized patients [1–3]. Lung ultrasound (LUS) has emerged as a reliable, non-invasive imaging tool for pneumonia diagnosis, with higher accuracy than chest radiography and second in sensitivity and specificity only to computed tomography (CT) [4–7]. While its diagnostic accuracy for pulmonary parenchymal alteration due to infection is established, the relationship between LUS findings and pneumonia etiology has not been clearly defined.

Conventional diagnostic tests (antigen detection, blood cultures, lower respiratory tract cultures, serology) identify pathogens in only 20–40% of cases, whereas testing with molecular syndromic panels (MSPs) based on multiplex PCR assays combined with cultures from bronchoalveolar lavage (BAL) achieve diagnostic yields of 90–95% [8].

This study aims to correlate LUS findings with microbiological and molecular results from BAL, considered the gold standard for etiological diagnosis. To our knowledge, this is the first study comparing LUS patterns of viral versus bacterial pneumonia in the Emergency Department (ED) using PCR- and culture-confirmed BAL results. Identifying etiology-specific sonographic patterns may facilitate rapid bedside differentiation of pneumonia in the ED, allowing timely initiation of targeted treatment and guiding further etiological investigations.

## Materials and methods

The study, titled “BAL-LUS Study, diagnostic accuracy of LUS in determining the aetiology of pneumonia in emergency settings: correlation between bronchoalveolar lavage findings and ultrasound signs” was approved by the Area Vasta Centro Ethics Committee (CEAVC 26198_oss) and registered on ClinicalTrials.gov (NCT06506617).

This is a non-profit, prospective observational study conducted on a cohort of patients with community-acquired pneumonia (CAP) and CAP with risk factors for multidrug resistant pathogens (CAP-MDR).

The study seeks to use high-quality respiratory samples (BAL) to correlate microbiological and molecular findings with LUS semiotics. BAL have been collected by bronchoscopy performed by emergency physicians and analysis with syndromic molecular panels and standard respiratory cultures, considered the gold standard in the etiological diagnosis of pneumonia.

### Study population and setting

The study was conducted in a hospital setting at the ED of the Careggi University Hospital (Firenze, Italy), an academic hospital serving as a local hospital for approximately 400,000 residents and acting as a referral center for nearly 1,600,000 inhabitants.

### Enrolment criteria

#### Inclusion criteria

The study enrolled adult patients (age >18 years) with a Rankin score < 5, who presented to the ED with a diagnosis of pneumonia. The diagnosis was confirmed based on clinical and radiological criteria defined by the IDSA guidelines (Infectious Disease Society of America) [1], which include: presence of a new pulmonary infiltrate on chest X-ray, with evidence of infectious origin with at least two of the following three clinical signs: fever >38 °C, leukocytosis or leukopenia and purulent respiratory secretions. To be included, the patients had to undergo BAL in the ED.

#### Exclusion criteria

The exclusion criteria for the study included: lack of informed consent, age < 18 years or > 90 years, pregnancy, life expectancy < 3 months, Rankin score ≥ 5, LUS not performed in all chest zones, pneumonia sustained by viral and bacterial coinfection and fungal pneumonia.

Patients with a Rankin score ≥ 5 were excluded, as severe baseline disability and comorbidities would confound pneumonia diagnosis and limit the feasibility of both bronchoscopy and multiplex PCR testing on BAL. In such cases, the risk–benefit ratio of performing an invasive procedure—including preparation, sedation, and execution—would not be justified, potentially exposing patients to higher procedural risks without proportional diagnostic advantage.

### Study objectives

The study aims to evaluate whether a correlation exists between the LUS semiotics and the etiopathogenetic agent (identified directly from BAL via molecular or traditional investigations) of pneumonia in patients presenting to the ED with pneumonia. Specifically, the study seeks to determine whether there are differences in the US imaging pattern presentation between bacterial and viral pneumonias.

### Diagnostic work-up

Patients presenting with respiratory or infectious symptoms undergo a standardized diagnostic work-up: this includes imaging studies, such as chest X-ray and computed tomography (CT), as well as laboratory investigations. As part of infection control and surveillance protocols, all patients with respiratory symptoms are also tested for SARS-CoV-2 via nasopharyngeal antigen swab, in accordance with institutional guidelines.

Subsequently, patients underwent BAL in ED for respiratory sample collection to obtain high-quality respiratory specimens, considered the gold standard for the etiological diagnosis of pulmonary infections.

### Bedside LUS

Patients underwent LUS performed by one of seven emergency physicians (sonographers). The sonographers had completed a certified ultrasound training program accredited by WINFOCUS or SIMEU and had independently performed at least 400 LUS examinations before the study started patients’ recruitment.

Point-of-care LUS was performed by sonographer following the initial clinical evaluation and history taking, and before chest radiography or CT. In cases where chest imaging preceded LUS, the ultrasound examination was conducted blinded to the radiological findings.

LUS was performed at the bedside using one out of 3 GE Vivid S5 ultrasound system (General Electric Healthcare, Milwaukee, WI, USA) equipped with a convex (2–5 MHz) probe, which served as the primary transducer for the examination. The thorax was systematically divided into 12 regions (anterior, lateral, and posterior for each hemithorax, further separated into superior and inferior regions). Each region was explored in longitudinal and oblique intercostal scans, with the probe oriented perpendicular to the pleural line. In cases where the convex probe did not allow optimal visualization of pleural or subpleural details, a high-frequency linear probe (7–12 MHz) was employed.

For lung ultrasound examinations, the convex probe depth was set between 6 and 10 cm, depending on patient body habitus, to include at least 2–3 cm of lung parenchyma below the pleural line. Gain was adjusted to optimize visualization of the pleural line and vertical artifacts, avoiding oversaturation, and the focal zone was positioned at the pleural line. For the linear probe, a shallower depth (approximately 3–5 cm) was used to enhance the resolution of the pleural line and interstitial changes; gain and focus were managed in the same manner.

Whenever possible, patients were assessed in the sitting position. In cases where this posture was not tolerated because of severe clinical status or limited cooperation, the examination was conducted in a supine or semi-recumbent position. Evaluation of the posterior fields was preferentially done with the patient seated; if this was not feasible, imaging was obtained by placing the patient in lateral decubitus on either side. Patients in whom a complete exploration of all pulmonary fields was not performed were excluded from the study.

All sonographic patterns were interpreted based on International Consensus Conference on Lung Ultrasound [9].

The sonographer recorded LUS findings on a standardized form for each of the 12 thoracic regions. Aeration patterns were classified as follows: normal aeration, with no more than two B-lines (A); mild loss of aeration, with ≥ 3 well-spaced B-lines in < 50% of the pleural surface (B1); moderate loss of aeration, with coalescent B-lines or involvement of > 50% of the pleural surface (B2); and consolidation, defined as a subpleural echo-poor area or one with tissue-like echotexture (C).

Consolidations > 1 cm were further categorized as Ca, if only static air or fluid bronchograms were present; Cb, if dynamic air bronchograms were identified; and Cc, if small subpleural hypoechoic consolidations measuring 0.5–1 cm were observed.

**Fig. 1:**
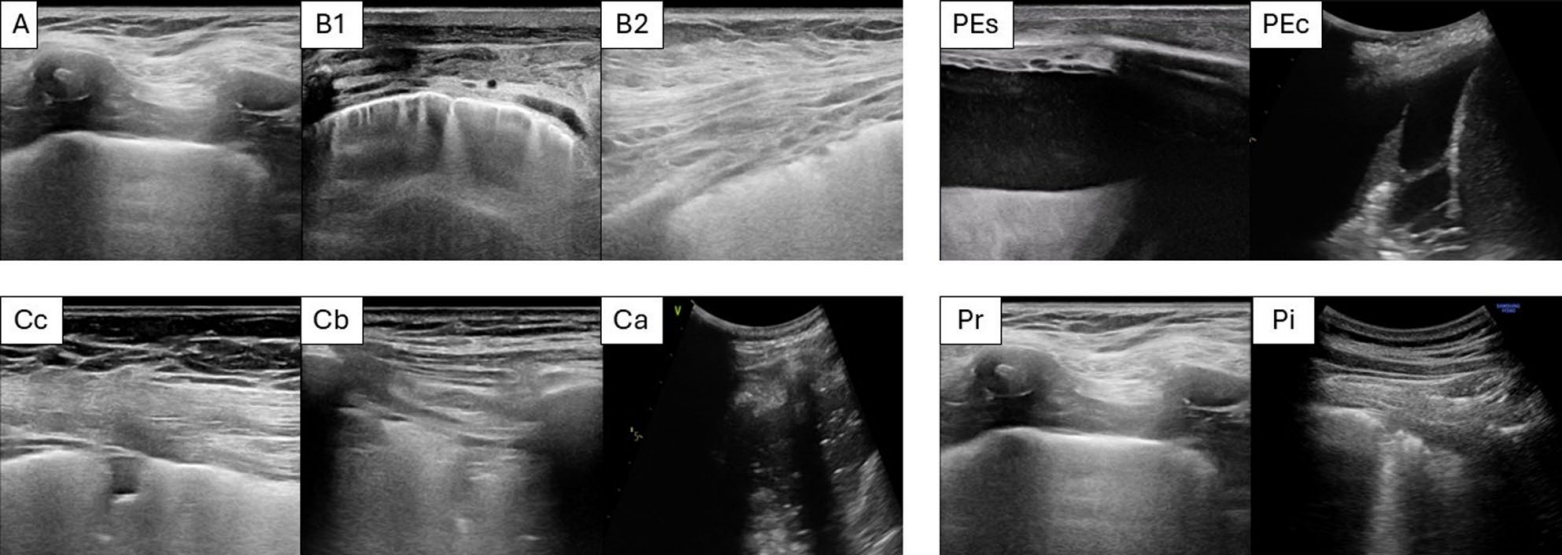
Lung ultrasound assessment. Left upper panels—from left to right: A pattern (A), at least more than 3 well-spaced B-lines visible in < 50% of the visualized pleura (B1), confluent B lines (B2). Right upper panels—from left to right: simple pleural effusion (PEs), complicated pleural effusion (PEc). Left lower panels—from left to right: small subpleural consolidation 0.5–1.0 cm in diameter (Cc), lung consolidation with dynamic bronchogram (Cb), lung fields with consolidation with no bronchogram or only static air or fluid bronchogram(s) (Ca). Right lower panels—from left to right: normal pleura (Pr), irregular pleura (Pi)

For each region, additional sonographic signs were reported, including irregular pleura (Pi), pleural effusion (PE), simple pleural effusion (PEs), and complex pleural effusion (PEc) (septations, echogenic debris, or a heterogeneous echotexture) (see Fig. 1 and standardized form in Supplementary Material). Findings were grouped by macro-region (anterior, lateral, posterior; superior vs. inferior) to evaluate the distribution of abnormalities.

A LUS score (range 0–36) was calculated according to the most severe aeration pattern detected in each region: score 0 = A; score 1 = B1; score 2 = B2; score 3 = C (including Ca or Cb). Patterns Cc, Pi, and PE (PEs or PEc) were not included in the score [10].

### Respiratory specimen collection

BAL sampling was performed in accordance with international recommendations, with bronchoscopic inspection guiding the procedure toward the bronchopulmonary segment most affected [11]. When chest CT was available, it was used to support localization; however, CT was performed only when clinically indicated. In patients without CT, the target site was identified through the integration of broncoscopic inspection and chest radiography findings [12, 13].

### Reference standard for diagnosis of bacterial vs. viral pneumonia (microbiological assessment)

Respiratory samples obtained by BAL were tested using an MSP (BIOFIRE^®^ FILMARRAY^®^ Pneumonia Plus Panel, bioMérieux) designed for hospital-acquired (HAP), ventilator-associated (VAP), and community-acquired pneumonia (CAP), targeting 18 bacterial pathogens, three atypical bacterial pathogens, nine respiratory viruses, and seven clinically-relevant resistance markers (REF).

Conventional culture was always performed in parallel according to standard protocols on non-selective media (blood, chocolate) and Sabouraud agar and incubated at 37 °C with 5% CO₂. Growth was evaluated at 36–48 h and colonies identified by MALDI-TOF MS. Results were expressed semi-quantitatively (CFU/mL). Antibiotic susceptibility testing was performed with automated platforms (BD Phoenix, TECAN).

Cases of fungal pneumonia were excluded from the study. Fungal cultures remain the reference standard but are slow and insensitive. To improve early detection, qualitative PCR assays on BAL allow rapid identification of Aspergillus spp., Pneumocystis jirovecii, and galactomannan. Serum or plasma biomarkers such as galactomannan and β-D-glucan (ELISA) provide indirect evidence of invasive fungal disease. While highly sensitive and useful in immunocompromised or critically ill patients, these assays were interpreted within the clinical and radiological context.

### Final diagnosis adjudication and follow-up

The final etiological adjudication (bacterial vs. viral and for excluding patients with coinfection and fungal infection) was independently performed by two senior emergency physicians with expertise in emergency medicine and infectious disease and in case of discordance the final adjudication was established by a third physician. They reviewed all available clinical and diagnostic data, including microbiological results from respiratory sample, medical documentation from the ED visit and any subsequent hospitalization, laboratory findings, imaging studies (including chest X-ray and CT scans where available). The final diagnostic adjudication was binary (viral or bacterial pneumonia). During final adjudication, patients with a clinical-radiological diagnosis of pneumonia were classified as having bacterial pneumonia if bacterial growth was detected on culture and/or if bacterial pathogens were identified through MSP testing in respiratory samples. Conversely, patients were classified as having viral pneumonia if a viral pathogen was qualitatively identified by MSP testing. According to the exclusion criteria, patients with no etiological diagnosis, fungal pneumonia or viral–bacterial coinfections were excluded. Coinfections may lead to an overlap between bacterial and viral forms, thus introducing potential confounding in the analysis, fungal pneumonia was excluded due to its relative rarity in this setting.

Patients were classified as having viral–bacterial coinfection when both pathogens were identified in the same individual. Fungal pneumonia was diagnosed in patients with positive culture results or when confirmed by one of the aforementioned methodologies.

### Statistical plan

The primary objective is to evaluate whether there is a correlation between a lung US parameter (continuous variable) and the etiological agent (dichotomous variable: bacterial vs. viral). To this end, the following statistical methods will be used: independent samples Student’s t-test, Mann–Whitney U test, and binary logistic regression.

The normality of variable distributions will be preliminarily assessed using Q–Q plots and the Shapiro–Wilk test. Differences in quantitative variables between two groups will be analyzed using either the parametric Student’s t-test or the non-parametric Mann–Whitney U test for variables without normal distribution. For comparisons involving more than two groups, ANOVA or the Kruskal–Wallis test will be employed, as appropriate. For qualitative variables, Fisher’s exact test will be applied.

The Receiver Operating Characteristic (ROC) curve was employed to assess the diagnostic performance of the continuous ultrasound variable in distinguishing between bacterial and viral pneumonia. Specifically, the area under the curve (AUC) was calculated as a measure of overall test accuracy.

Comparisons of mean LUS scores between viral and bacterial pneumonia were performed using Welch’s t-test and Mann–Whitney U test. Diagnostic performance was assessed by ROC curve analysis with calculation of the area under the curve (AUC) and optimal threshold based on the Youden index, with corresponding sensitivity and specificity. For the analysis of the regional distribution of ultrasound findings, proportions of each alteration in viral versus bacterial pneumonia were compared using Fisher’s exact test and chi-square test.

A two-tailed p-value of < 0.05 will be considered statistically significant. All statistical analyses will be performed using SPSS software (version 2019).

## Results

### Study population

**Fig. 2:**
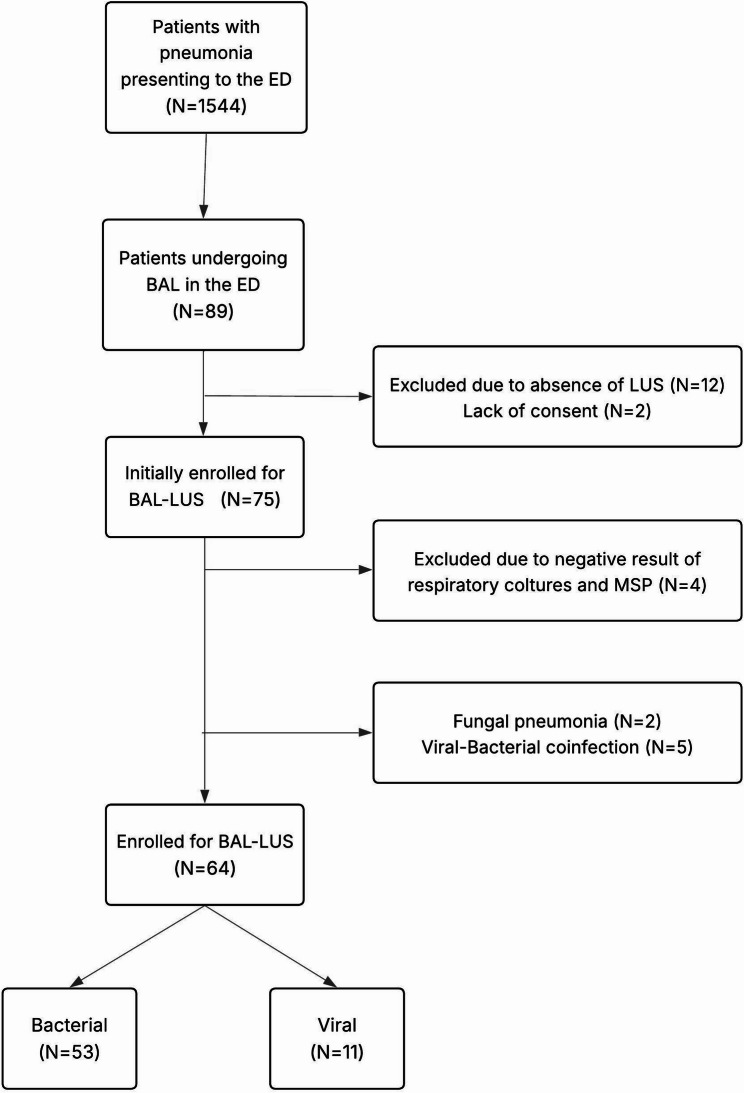
BAL-LUS workflow. Patients undergo BAL if they have PIRO > 2, moderate-to-severe acute respiratory distress syndrome (ARDS), septic shock from pulmonary source non-invasive ventilation or orotracheal intubation. LUS: lung ultrasound; MSP: Syndromic Molecular Panel. Twelve patients were excluded because LUS absence: LUS could not be performed at the time of evaluation due to technical and temporary logistical constraints

Between 1st June 2024 and 1st May 2025, a total of 75 patients presenting to the ED with pneumonia and fulfilling the study’s inclusion criteria were initially enrolled (Fig. 2). Of these, four were excluded due to the absence of an etiological diagnosis, two due to fungal pneumonia, and five due to viral–bacterial coinfection, with no patient excluded due to inability to complete the 12 regions LUS or BAL, leaving 64 patients definitively included. Among them, 11 cases were of viral etiology and 53 of bacterial origin. Females accounted for 45.3% of the enrolled population. The mean age of the patients included in the study was 73.3 ± 14.6 years.

No patients in dialysis, or patients with acute or symptomatic chronic heart failure, or pulmonary fibrosis, conditions potentially affecting LUS findings were enrolled.

**Table 1:** Population characteristics

	General population (*N* = 64)	Viral (*n* = 11)	Bacterial (*n* = 53)	*p*
Age	73.3 ± 14.6	66.4 ± 14.7	74.8 ± 14.3	0.88
Female sex	29/64 (45.3%)	6/11 (54.5%)	23/53 (43.4%)	0.36
Arterial hypertension	36/64 (56.2%)	6/11 (54.5%)	30/53 (56.6%)	0.58
COPD	27/64 (42.2%)	2/11(18.2%)	25/53 (47.2%)	0.07
TDM2	11/64 (17.2%)	0/11 (0.0%)	11/53 (20.8%)	0.10
Asthma	3/64 (4.7%)	1/11 (9.1%)	2/53 (3.8%)	0.44
Heart failure	10/64 (15.6%)	0/11 (0.0%)	10/53 (18.9%)	0.13
CAD	13/64 (20.3%)	1/11 (9.1%)	12/53 (22.6%)	0.29
Bronchiectasys	4/64 (6.2%)	1/11 (9.1%)	3/53 (5.7%)	0.54
Atrial Fibrillation	15/64 (23.4%)	2/11 (18.2%)	13/53 (24.5%)	0.49
DVT/PEm	6/64 (9.4%)	0/11 (0.0%)	6/53 (11.3%)	0.31
Stroke/TIA	8/64 (12.5%)	0/11 (0.0%)	8/53 (15.0%)	0.41
CKD	11/64 (17.2%)	1/11 (9.1%)	10/53 (18.9%)	0.39
Cirrosis and liver diseases	3/64 (4.7%)	0/11 (0.0%)	3/53 (5.7%)	0.56
Immunodepression	14/64 (21.9%)	5/11 (45.5%)	9/53 (17.0%)	**0.05**
Cognitive impairment	26/64 (40.6%)	3/11 (27.3%)	23/53 (43.4%)	0.26
Active cancer	14/64 (21.9%)	2/11 (18.2%)	12/53 (22.6%)	0.55

*COPD* Chronic Obstructive Pulmonary Disease, *TDM2* Type 2 Diabetes Mellitus, *CAD* Coronary Artery Disease, *DVT* Deep Venous Thrombosis, *PEm* Pulmonary Embolism, *TIA* Transient Ischemic Attack, *CKD* Chronic Kidney Disease

**Table 2:** Vitals and laboratory values

	General population (*N* = 64)	Viral (*n* = 11)	Bacterial (*n* = 53)	*p*
*Vitals*				
HR	94.0 ± 21.0	99.2 ± 13.9	93.0 ± 22.1	**0.03**
SAP	127.4 ± 29.7	121.6 ± 27.6	128.6 ± 30.2	0.84
DAP	71.5 ± 16.5	70.4 ± 17.7	71.7 ± 16.4	0.42
SpO2	90.9 ± 7.9	92.4 ± 6.5	90.5 ± 8.2	0.30
FiO2	33.8 ± 19.0	34.6 ± 25.6	33.6 ± 17.6	0.16
GCS	14.4 ± 1.9	14.9 ± 0.3	14.3 ± 2.1	0.09
BT °C	37.3 ± 1.1	37.8 ± 1.3	37.2 ± 1.1	0.29
*Arterial blood gas*				
pH	7.3 ± 0.9	7.5 ± 0.1	7.3 ± 1.0	0.44
pO2	64.1 ± 23.8	58.6 ± 9.6	65.2 ± 25.7	0.08
pCO2	39.7 ± 11.5	36.3 ± 9.1	40.4 ± 11.9	0.30
Lactate	1.9 ± 1.9	1.4 ± 1.2	2.0 ± 2.0	0.58
HCO3-	24.8 ± 6.4	25.2 ± 3.3	24.7 ± 6.9	0.20
Glucose	130.2 ± 49.1	114.2 ± 26.8	133.5 ± 52.1	0.13
Horowitz Index	229.7 ± 83.9	260.7 ± 82.1	223.3 ± 83.6	0.73
*Laboratory values*				
WBC *10^9^	12.3 ± 7.7	8.3 ± 7.1	13.2 ± 7.6	0.46
Hb g/dl	11.9 ± 2.2	12.2 ± 2.1	11.9 ± 2.3	0.82
Hct %	36.1 ± 7.1	36.5 ± 5.4	35.9 ± 7.4	0.33
Platelet	282.3 ± 121.0	201.4 ± 87.8	299.1 ± 120.7	0.09
Lymphocyte	0.9 ± 0.8	0.9 ± 0.7	0.9 ± 0.8	0.22
CRP	125.7 ± 131.8	70.1 ± 67.8	137.2 ± 139.2	**0.03**
PCT	6.4 ± 19.3	0.3 ± 0.4	7.6 ± 21.0	**0.04**
NT-proBNP	5802.4 ± 13788.3	391 ± 967.9	6925.5 ± 14922.2	**0.01**
Creatinine	1.2 ± 0.8	0.9 ± 0.4	1.3 ± 0.8	**0.05**
BUN	0.3 ± 0.5	0.1 ± 0.1	0.3 ± 0.6	**< 0.001**
INR	1.3 ± 0.6	1.4 ± 0.8	1.3 ± 0.6	0.42
ALT	41.3 ± 70.9	42.2 ± 59.2	41.2 ± 74.6	0.61
Bilirubin	0.6 ± 0.5	0.7 ± 0.4	0.6 ± 0.5	0.91
Na+	139.6 ± 6.1	141.1 ± 7.0	139.3 ± 5.9	0.82
K+	4.3 ± 0.6	4.1 ± 0.4	4.4 ± 0.6	0.41

*HR* Heart Rate, *SAP* Systolic Arterial Pressure, *DAP* Diastolic Arterial Pressure, *SpO₂* Peripheral Oxygen Saturation, *FiO₂* Fraction of Inspired Oxygen, *GCS* Glasgow Coma Scale, *BT* Body Temperature, *WBC* White Blood Cell Count, *Hb* Hemoglobin, *Hct* Hematocrit, *CRP* C-Reactive Protein, *PCT* Procalcitonin, *NT-proBNP* N-terminal pro-B-type Natriuretic Peptide, *BUN* Blood Urea Nitrogen, *INR* International Normalized Ratio, *ALT* Alanine Aminotransferase, *Na*^+^ Sodium, *K*^+^ Potassium

Following ED evaluation, 37 of 64 patients (57.8%) were admitted to the internal medicine ward, 22 (34.4%) to the sub-intensive care unit, and 5 (7.8%) to the intensive care unit. The 30-day mortality rate was 25.0% (16 of 64).

Regarding chest X-ray, 10 of 64 patients (15.6%) did not undergo the examination, 4 (6.2%) were negative for pneumonia, and 50 (78.2%) were positive for pneumonia. Chest CT was not performed in 24 patients (37.5%), while 40 (62.5%) identified alterations consistent with pneumonia; no patient who underwent CT had a negative image feature for pneumonia.

The baseline clinical characteristics of the overall population and the two subgroups are summarized in Table 1. The most frequently reported comorbidities were arterial hypertension (56.2%), COPD (42.2%), and cognitive impairment (40.6%) (Table 1). No significant differences were observed in comorbidities, except for immunodepression.

Table 2 reports the vital signs recorded at ED admission and laboratory test results performed in ED. Statistically significant differences were observed for heart rate, CRP, procalcitonin, NT-proBNP, creatinine and BUN.

### Microbiological testing

#### MSP testing

The pathogens detected using the MSP testing are reported in eTable—supplementary material. The panel yielded positive results in 93.75% of cases (60/64), with polymicrobial flora identified in 39 patients (60.9%).

The most frequently detected pathogens were *Staphylococcus aureus* (*n* = 17), *Klebsiella pneumoniae* (*n* = 16), *Haemophilus influenzae* (*n* = 12), and *Influenza A virus* (*n* = 10).

The table presents also the resistance genes identified using the MSP. The most frequently detected resistance gene was CTX-M, in combination with KPC (4/15, 26.7%).

#### Conventional culture

BAL culture tested positive in 60.9% (39/64) of patients, with evidence of multidrug-resistant (MDR) pathogens in 19 cases (29.6%).

The most frequently isolated organisms were* Klebsiella pneumoniae*,* Pseudomonas aeruginosa*, and* Staphylococcus aureus* (eTable—supplementary material).

### Study objective

Regarding the correlation between LUS findings and pneumonia aetiology, viral cases involved a higher number of lung fields and showed more extensive interstitial patterns, while bacterial pneumonias were more frequently associated with consolidations (C), intended as Ca or Cb, and PE (Table 3). Viral pneumonias also demonstrated greater prevalence of non-confluent (B1) and confluent B-lines (B2), and small subpleural consolidations (Cc) (Table 3). By contrast, bacterial cases were more often associated with consolidations displaying dynamic air bronchograms (Cb).

**Table 3:** Lung ultrasound features in viral and bacterial pneumonias

	Viral (*N* = 11)	Bacterial (*N* = 53)	*p*
N°	64.2 ± 17.6	75.5 ± 31.1	0.07
Lung field involved	6.1 ± 3.2	3.7 ± 2.3	**0.05**
B (B1 and B2)	4.9 ± 3.3	0.5 ± 1.3	**< 0.01**
B1	3.8 ± 3.0	0.4 ± 0.8	**< 0.01**
B2	1.2 ± 1.2	0.2 ± 0.7	**< 0.01**
C (Ca and Cb)	1.5 ± 0.9	2.9 ± 2.2	**< 0.01**
Ca	0.5 ± 1.8	0.5 ± 1.4	0.74
Cb	0.4 ± 1.0	2.5 ± 2.0	**0.03**
Cc	0.9 ± 1.8	0.2 ± 0.6	**0.01**
Pi	0.4 ± 1.2	0.5 ± 1.0	0.85
PE	0.3 ± 0.6	0.9 ± 1.3	**< 0.01**
Localization			
Right lung	3.1 ± 2.1	1.9 ± 1.4	**0.03**
Left lung	3.1 ± 1.7	1.9 ± 1.7	0.45
Anterior fields	1.9 ± 1.4	0.6 ± 1.0	**0.04**
Lateral fields	1.7 ± 1.4	1.2 ± 0.9	0.10
Posterior fields	2.6 ± 1.3	1.9 ± 1.0	0.11
Superior fields	2.9 ± 1.9	0.8 ± 1.3	0.07
Inferior fields	3.4 ± 1.6	3.0 ± 1.5	0.78

N°: total number of lung fields with any abnormality; B: number of lung fields with B-line pattern (B1 or B2); B1: number of lung fields with mild loss of aeration, at least 3 well-spaced B-lines visible in < 50% of the visualized pleura; B2: number of lung fields with moderate loss of aeration, coalescent B-lines or B-lines > 50% of visualized pleura C: number of lung fields with consolidation pattern (Ca or Cb); Ca: number of lung fields with consolidation with no bronchogram or only static air or fluid bronchogram(s); Cb: number of lung fields with consolidation with dynamic air bronchogram; Cc: number of lung fields with small (between 0.5 and 1 cm) subpleural hypoechoic consolidations without bronchogram; Pi: number of lung fields with irregular pleural line; PE: number of lung fields with pleural effusion

**Fig. 3:**
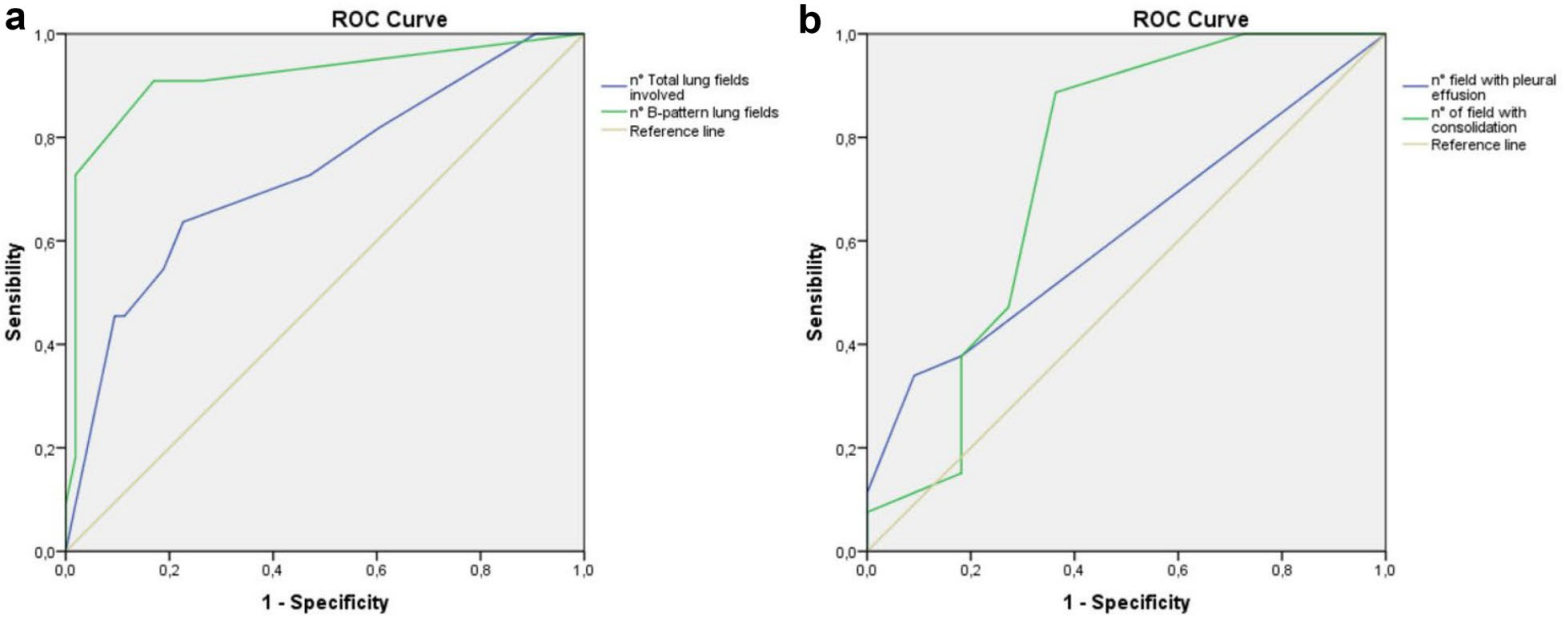
**a** Left panel: ROC curve of total lung fields involved and number of B-pattern (B1 or B2) lung fields in viral pneumonia. **b** Right panel: ROC curve of the number of pleural effusion fields and consolidation fields (Ca or Cb) in bacterial pneumonia

To explore the diagnostic accuracy of these features, ROC curve analysis was performed. An increasing number of involved lung fields correlated with viral etiology (AUC 0.73, 95% CI 0.55–0.90), with even higher accuracy for the number of B-line–positive fields (AUC 0.91, 95% CI 0.80–1.00) (Fig. 3a). For bacterial pneumonia, the number of consolidations and pleural effusion fields showed moderate discriminatory ability (AUC 0.74, 95% CI 0.53–0.94, and 0.62, 95% CI 0.45–0.78, respectively) (Fig. 3b).

**Fig. 4:**
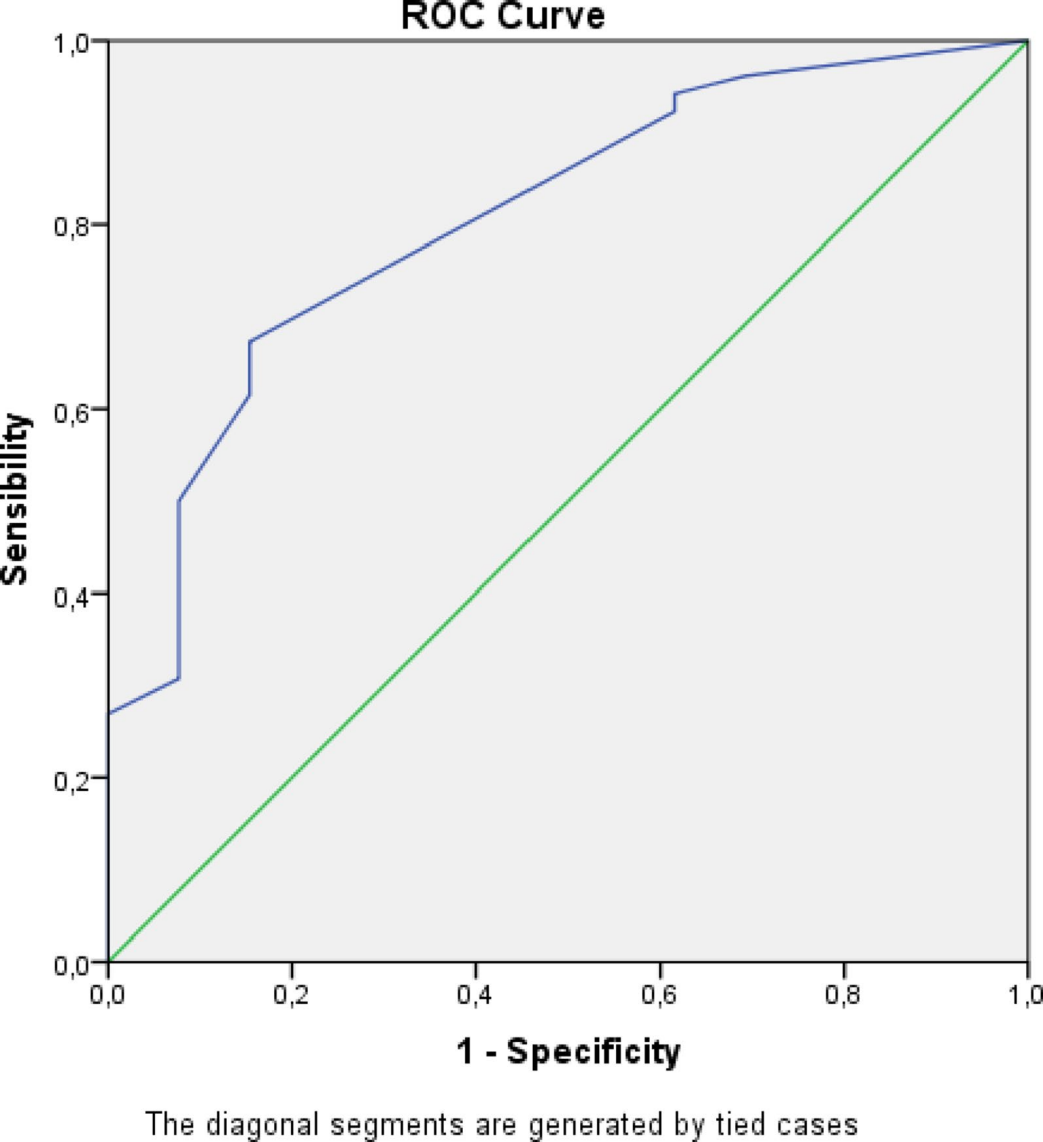
ROC curve of LUS score for bacterial vs. viral pneumonia

The mean LUS score was higher in bacterial than viral pneumonia (12.00 ± 6.28; 95% CI, 10.38–13.62; VS 5.86 ± 2.96; 95% CI, 4.15–7.56), with a statistically significant difference (Welch t-test *p* = 2.0 × 10⁻⁶; Mann–Whitney *p* = 2.32 × 10⁻⁴). The score showed good discrimination for bacterial vs. viral etiology (AUC = 0.81, 95% CI 0.68–0.93, *p* < 0.001) (Fig. 4).

### Regional distribution of LUS findings

When LUS alterations were analyzed by anatomical region (Table 3), relevant differences emerged between viral and bacterial pneumonia. When LUS findings were analyzed by anatomical region, several significant differences emerged between viral and bacterial pneumonia. The pattern of moderate B1 was consistently more frequent in viral cases, particularly in anterior and superior regions (e.g., anterior-superior right: 6/11 [54.5%] vs. 2/54 [3.7%], *p* = 0.0001; anterior-inferior right: 4/11 [36.4%] vs. 1/54 [1.9%], *p* = 0.002; anterior-superior left: 4/11 [36.4%] vs. 0/54 [0%], *p* < 0.001; lateral-superior right: 3/11 [27.3%] vs. 0/54 [0%], *p* = 0.004), and also in posterior fields (posterior-superior right: 5/11 [45.5%] vs. 1/54 [1.9%], *p* < 0.001; posterior-superior left: 4/11 [36.4%] vs. 2/54 [3.7%], *p* = 0.006). Conversely, consolidations with dynamic air bronchogram (Cb) were significantly more common in bacterial pneumonia, especially in posterior-inferior regions (posterior-inferior right: 34/54 [63.0%] vs. 1/11 [9.1%], *p* = 0.0017; posterior-inferior left: 30/54 [57.4%] vs. 1/11 [9.1%], *p* = 0.006). Severe B-lines (B2) and small subpleural consolidations (Cc) were also more frequent in viral pneumonia in posterior-inferior areas (B2: posterior-inferior left 4/11 [36.4%] vs. 2/54 [3.7%], *p* = 0.006; small subpleural consolidations: posterior-inferior left 3/11 [27.3%] vs. 2/54 [3.7%], *p* = 0.031). No significant differences were observed for lobar consolidations, pleural irregularities, or pleural effusions across regions.

**Fig. 5:**
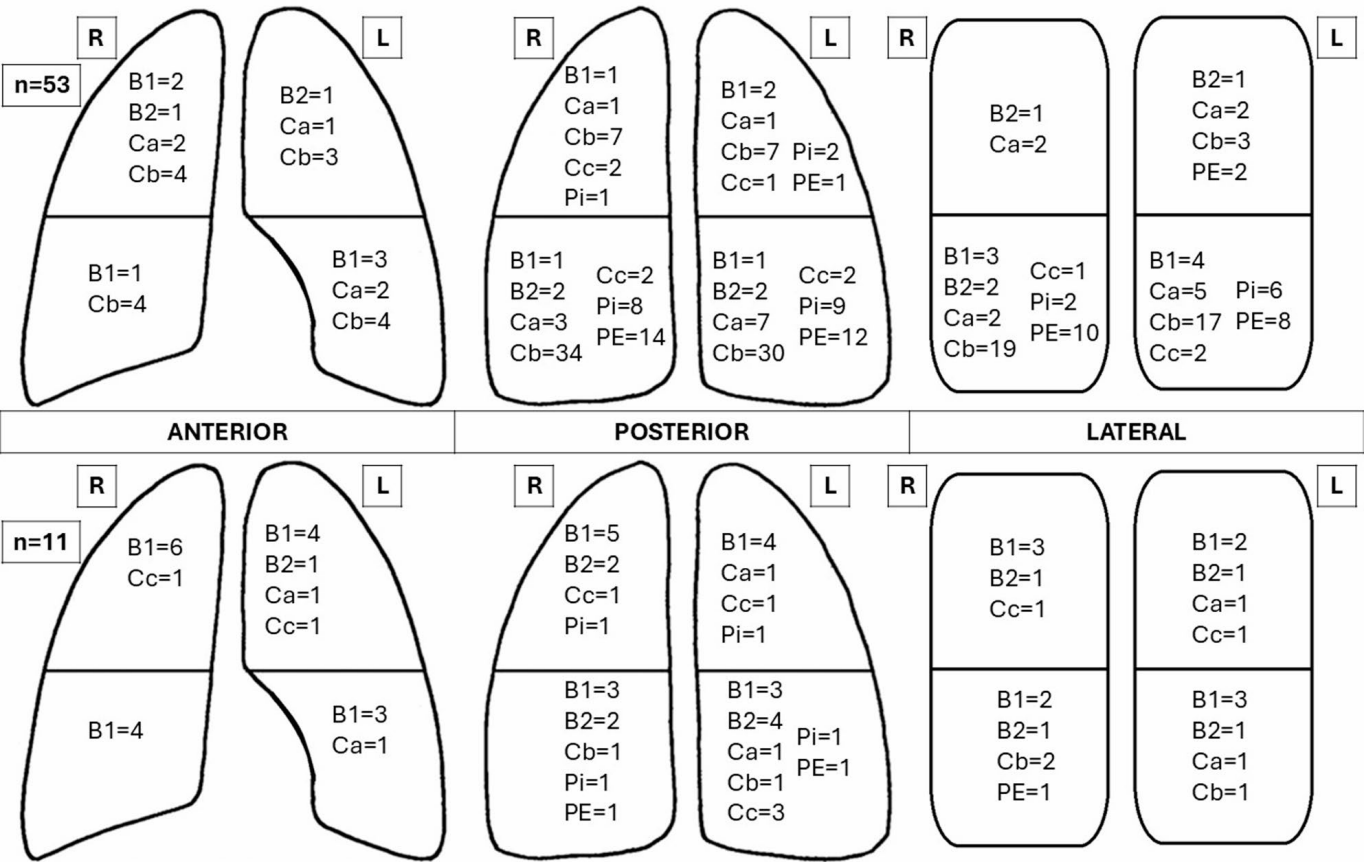
Alterations in each lung field, divided into bacterial pneumonia (upper panel) and viral pneumonia (lower panel). B1: mild loss of aeration, at least 3 well-spaced B-lines visible in < 50% of the visualized pleura; B2: moderate loss of aeration, coalescent B-lines or B-lines > 50% of visualized pleura; Ca: consolidation > 1 cm with no bronchogram or only static air or fluid bronchogram(s) ; Cb: consolidation > 1 cm with dynamic air bronchogram(s); Cc: small (from 0.5 to 1 cm) subpleural hypoechoic consolidations without bronchogram; Pi: irregular pleural line; PE: pleural effusion*. *No PEc was observed

The involved lung regions are reported in Fig. 5.

## Discussion

To our knowledge, this is the first study to correlate LUS findings with gold-standard etiological diagnoses obtained from BAL analysed by both MSP and cultures testing in the ED setting. While previous research has linked sonographic consolidations with bacterial pneumonia or relied on less accurate reference standards such as serology or nasopharyngeal swabs, our study directly compares LUS patterns with high-quality microbiological evidence.

Our findings suggest that LUS may provide supportive information to differentiate viral from bacterial pneumonia in emergency settings, although the observed differences should therefore be interpreted with caution, due to the presence of only borderline statistical significance in some cases, especially regarding the number of lung field involved. Viral infections were associated with broader lung involvement, interstitial syndrome, and small subpleural consolidations, whereas bacterial infections were characterized by larger consolidations and pleural effusion. These results align with prior paediatric studies, where the use of LUS for etiological discrimination is more established [14–16].

For example, Malla et al. demonstrated that bacterial pneumonia in children typically presents with hypoechoic consolidations and air bronchograms, while viral pneumonia is associated with B-lines and small subpleural consolidations, with reported sensitivity and specificity exceeding 90% [15]. Stoicescu et al. similarly found that LUS scores based on B-lines and consolidations differentiated bacterial from viral pneumonia with high accuracy [16]. Our data extend these observations to an adult emergency population, confirming the relevance of such sonographic features in real-world ED practice.

Applying a standardized 12-region LUS score corroborated the observed association between LUS patterns and BAL-confirmed etiology, yielding significantly higher scores in bacterial pneumonia and good discrimination (AUC = 0.814).

The higher frequency of small subpleural consolidations (from 0.5 to 1 cm) observed in viral pneumonia can be explained by the predominant involvement of the alveolar–interstitial interface in viral infections. Inflammatory changes and interstitial edema in areas adjacent to the pleura may lead to patchy alveolar collapse and the appearance of small consolidations directly abutting the pleural line. These subpleural consolidations are typically small (< 1 cm), multiple, and often bilateral, in contrast with the larger, lobar consolidations usually associated with bacterial pneumonia. This interpretation is consistent with several studies reporting the frequent presence of small subpleural consolidations in viral pneumonia, particularly in COVID-19 cohorts [17–20].

During the COVID-19 pandemic, several groups explored the value of LUS in distinguishing SARS-CoV-2 pneumonia from other aetiologies. The role of LUS in the first diagnostic approach to suspected COVID-19 pneumonia has been well established in international studies [21, 22]. Some clusters of interstitial and consolidation signs and their distribution became soon a diagnostic marker of patients admitted with high suspicion of the infection in a moment of very high incidence of the disease [23]. Tung-Chen et al. also reported that confluent B-lines and small subpleural consolidations were typical of COVID-19, while bacterial CAP more often showed hepatization and pleural effusion [24]. Bianchi et al. further validated the ability of LUS to stratify patients based on six reproducible patterns, achieving a predictive value above 95% [25]. These findings reinforce the potential of LUS as a non-invasive tool to support rapid diagnostic decisions across diverse respiratory infections, but the main evidence was obtained during a pandemic where the viral infections were largely prevalent.

Although our ROC analysis demonstrated good diagnostic capability of LUS alone, the moderate AUC values suggest that greater accuracy might be achieved by integrating ultrasound-derived variables with clinical and laboratory data readily available in the ED [26]; future studies with larger cohorts should address this need.

A strength of our study is that it was conducted during a period distant from the high incidence of viral pulmonary infections observed in previous pandemic waves. In our study, bacterial pneumonia was also associated with higher CRP, PCT, NT-proBNP, creatinine, and urea values, reflecting the greater inflammatory and systemic burden of bacterial infections. While the diagnostic utility of single biomarkers remains limited. PCT, for instance, has only moderate sensitivity and specificity [14, 27, 28], our results suggest that integrating LUS with laboratory markers such as PCT may improve diagnostic accuracy [27]. In their research, Omran et al. similarly demonstrated that combining LUS with the neutrophil-to-lymphocyte ratio enhanced discrimination between viral and bacterial pneumonia [29]. Such multimodal approaches may represent a pragmatic way forward in ED settings.

The etiological distribution observed in our cohort, with a predominance of Staphylococcus aureus and Klebsiella pneumoniae and a relatively low frequency of Streptococcus pneumoniae, reflects the changing epidemiology of CAP [30–33]. This shift is consistent with recent literature, where widespread pneumococcal vaccination and the increasing use of molecular diagnostics have revealed a more complex microbial landscape, with greater detection of viral pathogens and a higher prevalence of Gram-negative bacilli and S. aureus, particularly in elderly, comorbid, or COPD patients [30–32].

Our findings should be interpreted in the context of clinical evaluation, which remains the cornerstone of pneumonia diagnosis and management in the emergency setting. LUS can enhance clinical judgment by providing rapid bedside information, especially when its patterns confirm a diagnostic suspicion, but it cannot replace a comprehensive assessment. Future applications of artificial intelligence, particularly those based on pleural surface analysis, may help overcome these current limitations and provide additional support to clinicians at the bedside.

### Study limitations

This study has several limitations. First, its single-center design and modest sample size reduce generalizability. Second, the study population largely comprised patients with moderate-to-severe pneumonia, which may not reflect the broader spectrum of disease encountered in the ED. A further limitation is that chest CT was not systematically performed in all patients, which ideally represents the best option for driving the BAL; however, BAL localization was guided by bronchoscopic inspection combined with CXR findings according to international guidelines.

A limitation of the present study is that the duration of symptoms prior to ED presentation was not systematically recorded, and atypical bacterial pneumonias were not represented in our cohort; both factors may have influenced the observed ultrasound patterns.

A further limitation is that the same clinician performed the LUS examination and participated in the clinical management of the patient, potentially introducing observer bias. However, this reflects real-world emergency practice, where complete blinding is rarely achievable.

Finally, the imbalance between bacterial and viral cases, even if reflects the standard epidemiology pneumonia restricts the strength of subgroup comparisons and may have introduced bias [34]. Future multicenter studies with larger, more balanced cohorts are needed to validate and extend our findings.

## Conclusions

In adult patients presenting to the ED with suspected pneumonia, LUS shows a correlation with infection etiology: viral pneumonia typically involved interstitial syndrome, and small subpleural consolidations, whereas bacterial pneumonia more frequently showed larger consolidations and pleural effusion.

Although preliminary, these findings support the potential role of LUS as a rapid, bedside tool to complement laboratory e radiology diagnostics and potentially guide early treatment decisions. Importantly, LUS findings should always be interpreted in conjunction with the clinical context, as bedside ultrasound cannot replace comprehensive clinical judgment. Larger studies are warranted to confirm diagnostic accuracy and assess integration into routine ED workflows.

## Supplementary Information

Below is the link to the electronic supplementary material.

Supplementary Material 1

Supplementary Material 2

Supplementary Material 3

## Data Availability

The data that support the findings of this study are not publicly available due to their containing information that could compromise the privacy of research participants but could available from LP upon reasonable request.

## Abbreviations

ALT: Alanine aminotransferase
ARDS: Acute respiratory distress syndrome
AST: Antibiotic susceptibility testing
AUC: Area under the curve
BAL: Bronchoalveolar lavage
BT: Body temperature
BUN: Blood urea nitrogen
CAD: Coronary artery disease
CAP: Community-acquired pneumonia
CAP-MDR: Community-acquired pneumonia due to multidrug-resistant pathogens
CKD: Chronic kidney disease
COPD: Chronic obstructive pulmonary disease
CRP: C-reactive protein
CT: Computed tomography
DAP: Diastolic arterial pressure
DVP: Deep vein thrombosis (possibile ambiguità:conferma contesto se intendevi altro)
ED: Emergency Department
FiO2: Fraction of inspired oxygen
FU: Follow-up
GCS: Glasgow Coma Scale
HAP: Hospital-acquired pneumonia
Hb: Hemoglobin
Hct: Hematocrit
HDU: High dependency unit
HR: Heart rate
IDSA: Infectious Diseases Society of America
INR: International normalized ratio
LUS: Lung ultrasound
MSP: Syndromic molecular panel
NT-proBNP: N-terminal pro-B-type natriuretic peptide
OTI: Orotracheal Intubation
PCR: Polymerase chain reaction
PCT: Procalcitonin
PE: Pleural effusion
PEm: Pulmonary embolism
ROC: Receiver operating characteristic
SAP: Systolic arterial pressure
SpO2: Peripheral capillary oxygen saturation
TDM2: Type 2 diabetes mellitus
TIA: Transient ischemic attack
US: Ultrasound
VAP: Ventilator-Associated pneumonia
WBC: White blood cell count
