# Supplementary material for: Lung ultrasound for etiological diagnosis of pneumonia in the emergency department: correlation with bronchoalveolar lavage results
Source: Ultrasound J. 2025 Nov 27;17:63. doi: 10.1186/s13089-025-00470-0 (PMC12660545; doi:10.1186/s13089-025-00470-0)
Supplement: Supplementary file 1 — Supplementary Material 1 [file 13089_2025_470_MOESM1_ESM.docx]

| **PATHOGENS AND RESISTANCE GENES IDENTIFIED - Biofire® Filmarray® Pneumonia Plus Panel** | | | | |
| --- | --- | --- | --- | --- |
| **PATHOGENS** | **% total patients (N=64)** | | **RESISTANCE GENES** | **% total patients (N=64)** |
| **Acinetobacter calcoaceticus-baumanii** | 4 (6,2%) | | **CTX-M** | 15 (23,4%) |
| **Enterobacter cloacae complex** | 7 (10,9%) | | **KPC** | 5 (7,8%) |
| **Escherichia coli** | 8 (12,5%) | | **mecA/C e MREJ** | 4 (6,2%) |
| **Coronavirus** | 4 (6,2%) | | **VIM** | 1 (1,6%) |
| **Haemophilus influenzae** | 12 (18,7%) | |  |  |
| **Klebsiella oxytoca** | 1 (1,6%) | |  |  |
| **Klebsiella pneumoniae** | 16 (25%) | |  |  |
| **Moraxella catarrhalis** | 4 (6,2%) | |  |  |
| **Proteus spp** | 3 (4,7%) | |  |  |
| **Influenza a** | 10 (15,6%) | |  |  |
| **Legionella pneumophila** | 1 (1,6%) | |  |  |
| **Pseudomonas aeruginosa** | 9 (14,1%) | |  |  |
| **Metapneumovirus** | 1 (1,6%) | |  |  |
| **Staphylococcus aureus** | 17 (26,6%) | |  |  |
| **Streptococcus pneumoniae** | 7 (10,9%) | |  |  |
| **Rhinovirus/Enterovirus** | 7 (10,9%) | |  |  |
| **PATHOGEN IDENTIFIED ON RESPIRATORY SAMPLE CULTURE** | | | | |
| **PATHOGEN IDENTIFIED** | n° (MDR) | **PATHOGEN IDENTIFIED** | | n° (MDR) |
| Klebsiella pneumoniae | 10 (6) | Klebsiella oxytoca | | 1 (0) |
| Pseudomonas aeruginosa | 6 (3) | Providencia stuartii | | 1 (1) |
| Staphylococcus aureus | 6 (1) | Streptococcus anginosus | | 1 (0) |
| Haemophilus influenzae | 6 (0) | Proteus mirabilis | | 1 (1) |
| Escherichia coli | 5 (3) | Enterobacter cloacae | | 1 (1) |
| Streptococcus pneumoniae | 3 (0) | Acinetobacter baumannii | | 1 (1) |
| Corynebacterium striatum | 2 (2) | Enterococcus faecium | | 1 (0) |

***eTable – Supplementary material.*** *Pathogens and resistance genes identified by Biofire Filmarray Pneumonia Plus Panel and respiratory sample culture*
