# Supplementary material for: Lung ultrasound for etiological diagnosis of pneumonia in the emergency department: correlation with bronchoalveolar lavage results
Source: Ultrasound J. 2025 Nov 27;17:63. doi: 10.1186/s13089-025-00470-0 (PMC12660545; doi:10.1186/s13089-025-00470-0)
Supplement: Supplementary file 2 — Supplementary Material 2 [file 13089_2025_470_MOESM2_ESM.docx]

**Supplementary Material - Criteria for Performing Bronchoalveolar Lavage (BAL) in Patients with Pneumonia**

BAL was performed in patients with a clinical and radiological diagnosis of pneumonia when at least one of the following conditions was met, according to institutional protocols and international bronchoscopy guidelines:

1. **Failure of initial empiric antibiotic therapy**, defined as lack of clinical or radiological improvement after ≥48–72 hours of appropriate antimicrobial treatment.
2. **Severe pneumonia at presentation**, including patients with:
   - respiratory failure requiring high-flow oxygen, noninvasive, or invasive ventilation;
   - hemodynamic instability or sepsis requiring vasoactive support;
   - multilobar or rapidly progressive infiltrates on imaging.
3. **Immunocompromised state**, such as:
   - hematologic malignancy or solid tumor under active chemotherapy;
   - solid-organ or bone marrow transplantation;
   - long-term corticosteroid or immunosuppressive therapy;
   - advanced HIV infection (CD4 <200 cells/μL).
4. **High suspicion of atypical, opportunistic, or nosocomial pathogens**, including multidrug-resistant (MDR) bacteria, mycobacteria, fungi, or viruses, based on clinical or radiological features.
5. **Need for microbiological confirmation before targeted therapy**, in patients at risk for MDR infection or when infection control measures were required.
6. **Radiological or clinical discordance**, when imaging findings (e.g., diffuse infiltrates, ground-glass opacities, or nodular patterns) did not correspond to the presumed etiology or clinical presentation.
7. **Airway obstruction or mucus impaction** suspected on imaging or bronchoscopy, requiring both diagnostic and therapeutic lavage.

BAL was not routinely performed in mild or moderate community-acquired pneumonia (CAP) with a clear clinical course and rapid response to empiric therapy.

The main criteria leading to BAL in ED were related to clinical or radiological severity; In particular, in our ED, BAL is typically performed only in selected patients meeting one or more of the following conditions: **severe pneumonia at presentation, immunocompromised state, high suspicion of atypical, opportunistic, or nosocomial pathogens, need for microbiological confirmation before targeted therapy, or airway obstruction or mucus impaction**.
