# Supplementary material for: Lung ultrasound for etiological diagnosis of pneumonia in the emergency department: correlation with bronchoalveolar lavage results
Source: Ultrasound J. 2025 Nov 27;17:63. doi: 10.1186/s13089-025-00470-0 (PMC12660545; doi:10.1186/s13089-025-00470-0)
Supplement: Supplementary file 3 — Supplementary Material 3 [file 13089_2025_470_MOESM3_ESM.docx]

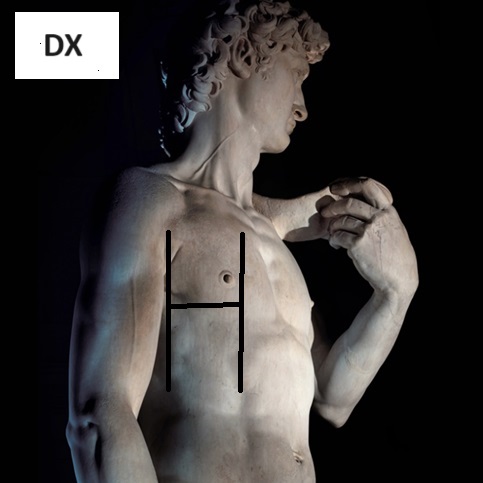

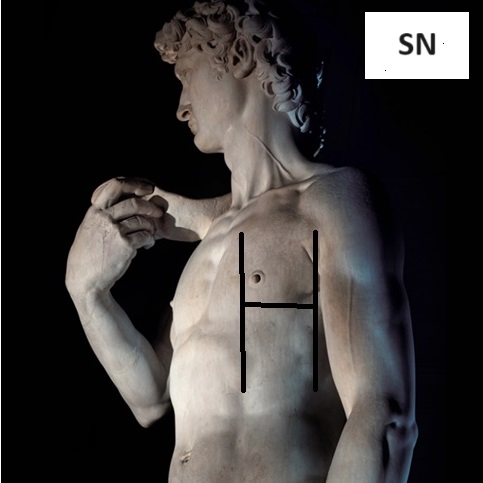

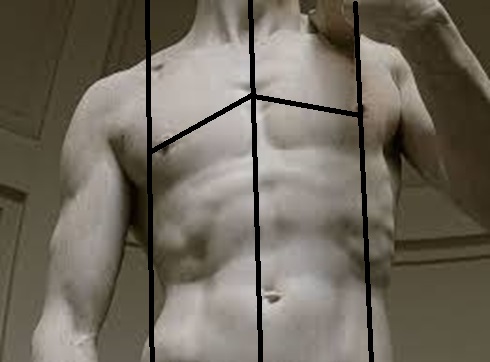

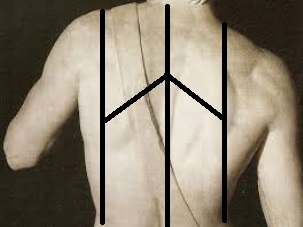


Legend for Lung Ultrasound Findings

1. Lung Characteristics

- Normal (A): normal aeration, with no more than two B-lines
- B1: mild loss of aeration, with ≥3 well-spaced B-lines in <50% of the pleural surface
- B2: moderate loss of aeration, with coalescent B-lines or involvement of >50% of the pleural surface.
- C (Consolidation: a subpleural echo-poor area or one with tissue-like echotexture):
  - Ca: Consolidations >1 cm if only static air or fluid bronchograms were present
  - Cb: Consolidations >1 cm and dynamic air bronchograms
  - Cc: small subpleural hypoechoic consolidations measuring 0.5–1 cm were observed.

2. Pleural Features

- Pr: Regular pleura.
- Pi: Irregular pleura.

3. Pleural Effusion (PE)

- PEs: Simple effusion.
- PEc: Complicated effusion (septations, echogenic debris, or a heterogeneous echotexture).
